# When Change Causes Stress: Effects of Self-construal and Change Consequences

**DOI:** 10.1007/s10869-015-9411-z

**Published:** 2015-07-05

**Authors:** Barbara Wisse, Ed Sleebos

**Affiliations:** Department of Psychology, Faculty of Behavioral and Social Sciences, University of Groningen, Grote Kruisstraat 2/1, 9712 TS Groningen, The Netherlands; Department of Organization Sciences, Faculty of Social Sciences, VU University Amsterdam, De Boelelaan 1081, 1081 HV Amsterdam, The Netherlands

**Keywords:** Organizational change, Self-construal, Personal identity, Collective identity, Uncertainty, Stress

## Abstract

**Purpose:**

Organizational change can be a major stress factor for employees. We investigate if stress responses can be explained by the extent to which there is a match between employee self-construal (in personal or collective terms) and change consequences (i.e., does the change particularly have consequences for the individual or for the group). We further investigate if the interactive effect of self-construal and change consequences on stress will be mediated by feelings of uncertainty.

**Design/Methodology/Approach:**

Data were obtained in three studies. Study 1, a laboratory study, focused on *physiological stress*. Study 2, a business scenario, focused on *anticipated stress*. Study 3, a cross-sectional survey, focused on *perceived stress*. Studies 2 and 3 also included measures of uncertainty in order to test its mediating qualities.

**Findings:**

Change is more likely to lead to stress when the change has consequences for matters that are central to employees’ sense of self, and particularly so when the personal self is salient. This effect is mediated by feelings of uncertainty.

**Implications:**

Understanding why some people experience stress during change, while others do so to a lesser extent, may be essential for improving change management practices. It may help to prevent change processes being unnecessarily stressful for employees.

**Originality/Value:**

This is one of the first studies to show that different kinds of change may be leading to uncertainty or stress, depending on employees’ level of self-construal. The multi-method approach boosts the confidence in our findings.

It has been suggested that the low success rate of organizational change may partly be explained by the fact that organizational change can take a huge toll on the employees (Fugate et al. 2012). Indeed, although (especially self-initiated) change can be a source of employee engagement (Bakker et al. 2012), organizational change processes often have a host of disruptive effects on employees (Bordia et al. 2004; Cartwright and Schoenberg 2006; Jimmieson et al. 2004; Oreg et al. 2011). The most notable effect of organizational change processes is that it frequently leads to employee stress. Support for this argument comes from several studies that have assessed how *perceptions* of stress (reported feelings of being overextended and depleted of one’s emotional and physical resources; Maslach et al. 2001) or *physiological* stress responses (indicating how stress affects bodily systems) are affected by organizational change. For instance, Johnson et al. (2006) found that with increasing levels of change, self-reported stress among staff members also increased (cf., Ashford 1988; Oreg et al. 2011). Furthermore, Dahl (2011) found that the risk of receiving stress-related medication (to combat anxiety attacks, insomnia, blood pressure—BP problems, etc.) increased significantly for employees in organizations that were undergoing change (cf., Greubel and Kecklund 2011).

However, there are large inter-individual differences in the amount of stress that employees experience as a result of change. Indeed, in contrast to the general belief that individuals are prone to react to change in a consistent manner, research indicates that people’s level of change-related distress varies according to the change incident (Bareil et al. 2007). Understanding why some people experience stress when facing a particular change, while others do not, or experience it to a lesser extent, may be essential for improving change management practices. For instance, it may help to prevent change processes being unnecessarily stressful for employees and to ensure that change agents do not miss opportunities to manage change effectively. Building on theory of work stress (Demerouti et al. 2001; Lazarus and Folkman 1984) and on the role of self-construal (Brewer and Gardner 1996; Eilam and Shamir 2005; Sani 2008), we argue that what particularly affects stress responses is change that is perceived to threaten what people deem important and thus makes them uncertain. Specifically, we will test the general proposition that the extent to which there is a match between employee self-construal (in personal or collective terms) and the consequences of the change (i.e., does the change impact individual functioning or group functioning) predicts stress responses. We thus expect that people will experience more stress when a change targets their salient level of self-construal. We further predict that the interactive effect of change consequences and self-construal on stress will be mediated by feelings of uncertainty (see Fig. 1).

**Fig. 1:**
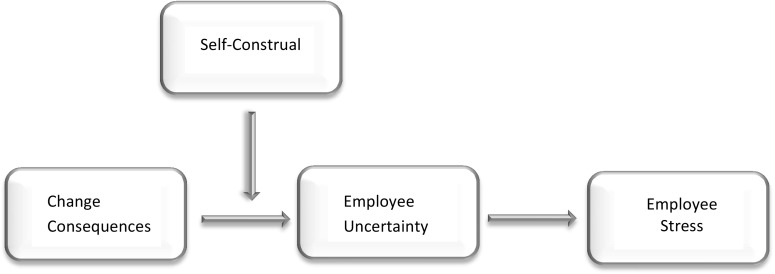
Research model depicting the proposed combined effects of self-construal and change consequences on employee uncertainty and stress

## Organizational Change Can be Demanding and Stressful

Most models of occupational stress posit that job demands (or a negative cognitive appraisal of an event or situation) negatively impact employee well-being and/or positively affect employee stress. For instance, the Job Demands-Resources model (JD-R; Demerouti et al. 2001; Schaufeli and Bakker 2004) argues that high job demands have energy-depleting properties and may elicit emotional exhaustion and ill health (Bakker et al. 2005). Job demands can be aspects of employment like work load and conflict, but organizational change can be a job demand as well (Nikolova et al. 2014). In line with these models, it has been found that organizational change processes often have a negative impact on how employees feel (Bordia et al. 2004; Cartwright and Schoenberg 2006; Jimmieson et al. 2004; Oreg et al. 2011). Importantly, according to the cognitive stress model of Lazarus and Folkman (1984), the appraisal of an event or the way in which an individual evaluates a situation may be more important to employee well-being and stress than the actual presence of stressors. This suggests that the impact of changes in the work environment on employee stress is dependent on the cognitive appraisal of these changes: some changes may be perceived as demanding and thus stressful to some people, while others may not. A key issue here is that not all changes are equally stressful to all people. We argue that it is particularly change that is perceived to threaten what is valuable to people that affects stress responses. A critical predictor of what people hold dearly and of how they will respond to their external environment, is how people construe their perceptions of the self. Therefore, we will turn our attention to self-construal in the following.

## The Role of the Self in Uncertainty and Stress as a Result of Change

The self can be understood as the way people perceive themselves or the knowledge they have about themselves. In essence, it provides an answer to the question ‘Who am I?’. The answer to this question fundamentally influences the way people feel, think, perceive, behave, and strive for particular goals (Leary and Tangney 2003; Oyserman and Lee 2008). As a consequence, the self is also of paramount importance to people’s functioning in organizational contexts.

The self can be construed at different *levels of inclusiveness* (Aron et al. 1991; Brewer and Gardner 1996; Sedikides and Brewer 2001). People may think about or perceive themselves in more personal terms or in more social terms. The personal form of self-construal is activated when an individual’s sense of unique identity and how he or she differentiates himself or herself from others, is the basis for self-definition (i.e., the *personal self*). At this level, personal self-related features are accentuated, and the influence of others in the self-schema is minimized (van Baaren et al. 2003). When the personal self is salient, people tend to strive for self-enhancement and to focus on self-benefit (Brewer and Gardner 1996; Lee et al. 2000; Utz 2004). Individuals with a salient personal self may thus particularly value those aspects of their job that allow them to focus on self-enhancement and grant them the opportunity to derive benefits for themselves (Brickson 2000). The self may, however, also be expanded to incorporate others. When the collective form of self-construal is activated (i.e., the *collective self* or social identity), the basis for self-definition is derived primarily from one’s group memberships. At this level, self-construal implies a psychological merging of self and group, which leads individuals to see the self as similar to other members of the collective and to ascribe group-defining characteristics to the self. When the collective self is salient, people tend to strive for collective welfare and group enhancement (Brewer and Gardner 1996; Lee et al. 2000; Turner et al. 1987; Utz 2004). Those who have a salient collective self may therefore particularly value aspects of the job that foster group enhancement and that provide opportunities for collective welfare (Ashforth et al. 2008).

The self is a relatively dynamic and complex concept: multiple self-construals can co-exist within one individual and can be activated at different times or in different contexts (van Baaren et al. 2003; Oyserman and Lee 2008). However, usually one of those levels tends to be more salient at a given point in time (Lord and Brown 2004). Notably, those salient elements have more influence on an individual’s cognitions, emotions, and behaviors than less salient elements of the self.

Given that what people value and strive for depends on their level of self-construal, one may argue that—depending on their level of self-construal—they will also feel threatened by different situations. In the case of organizational change, this implies that those with a salient personal self will be threatened by change that affects their goals of self-enhancement and self-benefit, while those with a salient collective self will be threatened by change that affects their goals of group enhancement and collective well-being. However, whichever level of self-construal is salient, the issue is that when change prevents employees from enacting their salient identity and threatens that which they hold most dear in their work, it will result in feelings of uncertainty (a sense of doubt, confusion, and unpredictability) that elicit stress responses. Several theoretical arguments in the literature on self and identity corroborate this assertion. For instance, Petriglieri (2011) argued that “experiences appraised as indicating potential harm to the value, meanings, or enactment of an identity” are aversive to people (p. 644). Indeed, people feel uncomfortable with situations that threaten their identity and they value a sense of self-continuity (a sense of stability in their self-perception over time and across situations; Sani et al. 2008). Moreover, it has been found that people who see a threat to the value, meanings, or enactment of their identity feel less certain about who they are, and as a consequence, they experience less subjective well-being (Ritchie et al. 2011).

Empirical findings from studies on reactions to change also corroborate our line of reasoning. For instance, it has been found that organizational change often evokes feelings of uncertainty in employees (Ashford 1988; Bordia et al. 2004), and that these, in turn, can elicit stress responses (Bordia et al. 2004; De Cuyper et al. 2010; Schabracq and Cooper 1998; Terry and Callan 1997). Testifying to the importance of the role of people’s self-concept in reactions to change is research that shows that when people do not have a sense of self-continuity, they find it more difficult to cope effectively with potentially stressful job-related events (Eilam and Shamir 2005; Sadeh and Karniol 2012). Moreover, it has been found that the more people see the merged group as a continuation of their pre-merger group (and thus feel their identity to be less threatened), the closer the association is between their pre-merger identification and their post-merger identification (Boen et al. 2007; van Knippenberg et al. 2002; Ullrich et al. 2005). In a similar vein, it has been suggested that job satisfaction and citizenship behavior are higher, and turnover intentions and negative emotions are lower, for employees who retain a sense of continuity during a merger process, than for those who do not (van Dick et al. 2004).

Missing in previous research is empirical evidence showing that different kinds of change may be regarded as discordant, depending on the level of self-construal. In addition, the few studies that employed a self-construal perspective on change did not focus on stress responses, which is unfortunate as this may be a key to gaining a clear understanding of change processes. The present research therefore focuses on the question of whether change that relates to the functioning of the group (e.g., group goals, composition, or identity) has different effects on employee stress than change that relates to the functioning of the individual (e.g., personal goals, characteristics or identity), depending on whether the personal or the collective self is salient. Finally, previous research did not investigate uncertainty as a potential mediator of the combined effect of self-construal and change consequences on stress. The current study is therefore geared at uncovering the mediating qualities of uncertainty. Our general hypotheses are the following:

### **Hypothesis 1**

People will experience more stress when a change targets their salient level of self-construal.

### **Hypothesis 2**

Feelings of uncertainty will mediate the interactive effect of the target of change and self-construal on stress.

## Overview of the Studies

In Study 1, a laboratory study, participants performed a task that required them to deal with customer requests. During the task, they were confronted with a change in performance measurement. We manipulated who would be most affected by the change (the individual vs. the group) and self-construal (personal vs. collective), and we measured physiological stress (i.e., BP). We then tested Hypothesis 1. Study 2, a scenario study amongst employees, involved a change in business strategy and goal orientation. We manipulated the consequences of the change and self-construal, and we measured uncertainty and anticipated psychological stress. We then tested Hypotheses 1 and 2. Finally, in Study 3, we used a sample of employees who were in the midst of an organizational change process. We measured personal self-construal, the extent to which the change was perceived to affect the individual and the group, uncertainty, and perceived stress. This allowed us to assess the generalizability of the findings of Study 1 and 2.

## Method Study 1

### Participants and Design

Eighty-eight Dutch students (28 males, *M*_age_ = 21.19, SD = 3.41), who participated voluntarily, were randomly assigned to a 2 (Self-Construal: personal vs. collective) × 2 (Change Consequences: individual vs. group) between-subjects design. The 4 conditions comprised between 20 and 24 participants each. A total of 92.1 % of our participants had held or held at the time a full-time or part-time job.

### Procedure

Participants were seated in one of five individual cubicles equipped with computers. All the information and measures were administered via the program software. Thus, participants’ opportunities to engage in visual contact and face-to-face communication with each other were limited, but they were led to believe that a network connection among them would be established. In reality, interaction was simulated via the experimental set-up. Participants were first exposed to our self-construal manipulation (Wisse and Rus 2012). Next, they were introduced to the main task. The task, adapted from Hertel et al. (2003), simulated a computer retail store in which participants had to process pre-programmed customer requests (cf. Damen et al. 2008). Specifically, participants had to put together hardware packages of consisting of a personal computer (PC), a monitor, and a printer according to customer requests. They were told that several aspects of task performance would be considered: the number of orders processed, number of mistakes (i.e., failures to match customer requests), profit (i.e., based on the total price of the hardware package), and pace (i.e., how well they were able to work at a steady and even tempo). Moreover, we told participants that scores for individual and group performance (based on the average individual scores) would be calculated (allegedly based on ‘norm-scores’ that were developed for the task) for each of the four criteria. These scores could range from 1 (very poor) to 10 (excellent). The task was to be performed 3 times (4 min per round), and feedback on individual and group performance was provided after the first two rounds. For the first two rounds, all aspects of the task were said to be equally important for the total score. Based on the total score, a spot on a top-score list could potentially be obtained. Importantly, we told participants that after round two, a group leader would be appointed. This person would be picked randomly out of the group of participants who were present in the lab. This leader would be given the opportunity to make strategic changes relating to the task. All the group leaders, who were in fact simulated by the computer software, communicated that changes in score calculation would be made. These changes had differential expected consequences depending on task performance in round 1 and 2. After conducting the task for the third and final time, participants were asked some more questions, then debriefed, thanked, and paid.

#### Self-construal Manipulation

The *self*-*construal manipulation* consisted of an experiential priming procedure (Wisse and Rus 2012; cf. Oyserman and Lee 2008). In the *personal self* condition, participants were asked to provide a written report on how they, as an individual, function when completing tasks and to recall a time when they had worked on a task independently. They also had to think about their personal goals, skills, and qualities that were relevant for accomplishing tasks. In the *collective self* condition, they were asked to write about how they, as a group member, function when completing tasks and to recall a time when they had worked on a task collectively. They also had to think about the group’s goals and their skills and qualities as members of the group that were relevant for accomplishing tasks.

#### Change Consequences Manipulation

After the second round of the main task, the leader announced that changes would be made to the score calculation. Specifically, the leader emailed all group members to say: “I, as leader, (…) consider the aspects profit and pace very important and I want you to pay more attention to those aspects. For that reason, I have decided that the scores for profit and pace will count double in the calculation of the total score. In the first two rounds of the order-processing task, all aspects were weighted equally for the total score. From now on, however, profit and pace will count double.” Importantly, depending on condition, these changes would most strongly affect the individual or the group.

Specifically, these differential effects were realized because in the *individual consequences* condition, participants had previously received feedback that their individual scores on the first and second rounds of the task were 8 and 9 for the number of processed orders, 8 and 8.5 for the number of mistakes, 5 and 4.5 for profit, and 5 and 5 for pace (thus relatively low individual scores were obtained on profit and pace). In contrast, the group scores on the first and second round of the task were 7 and 7.5 for the number of processed orders, 6 and 6 for the number of mistakes, 7 and 7 for profit, and 6 and 6.5 for pace (thus, the group scores on profit and pace were not necessarily lower than those for number of orders and mistakes). Clearly, in this condition, the changes would have more severe performance consequences for the individual than for the group.

In the *group consequences* condition, the feedback was the mirror image of that used for the individual feedback condition so that the changes in the scoring system would have more severe consequences for group task performance than for individual task performance. That is, group scores on rounds 1 and 2 were, respectively, 8 and 9 for the number of processed orders, 8 and 8.5 for the number of mistakes, 5 and 4.5 for profit, and 5 and 5 for pace (thus, relatively low group scores were obtained on profit and pace). In contrast, the individual scores on rounds 1 and 2 were, respectively, 7 and 7.5 for the number of processed orders, 6 and 6 for the number of mistakes, 7 and 7 for profit, and 6 and 6.5 for pace (thus, the individual scores on profit and pace were not necessarily lower than those for number of orders and mistakes). Note that in both conditions, the total scores for the individual and the group were equal.

### Dependent Measures

In order to assess the success of the *self*-*construal manipulation*, we asked participants to indicate whether at the beginning of the experiment—where they had typed in a description of their ideas, opinions, and thoughts about task performance—they recalled how they individually function during task performance or how they perform as a group member during task performance (1 = *how I perform during individual tasks*; 2 = *how I function during team or group tasks*). As a *change consequences* manipulation check, we used the average score of participants’ agreement with two statements (1 = *strongly disagree*; 7 = *strongly agree*): “The change threatened my personal functioning,” and “The change could particularly negatively affect my personal functioning” (*α* = .72).

As an indicator of *physiological stress,* we used a so-called ‘home BP measure.’ BP changes stem from blood flowing from the heart and/or resistance in the arteries. It is measured at two points: the systole, the point at which the force exerted by the blood on artery walls is greatest, and the diastole, the point at which the blood exerts the least force on the artery walls. These two measurements are known as systolic blood pressure (SBP) and diastolic blood pressure (DBP). We used a non-invasive monitor (model BraunBP1650) to record SBP and DBP from the radial artery of participants’ right arm twice before and twice after the change in scoring was announced.^1^ We averaged the SBP measures of the two recordings before the announcement, and we averaged the SBP measures of the two recordings after the announcement. We followed the same procedure for DPB. It should be noted that SBP is more sensitive to change-related stress (Pollard 2001) in the short term. Because in our experiment, participants would have had only a relatively short exposure to the stressor at the point when the measurements were taken, we anticipated that there would be fluctuations only in SBP.

## Results Study 1

In all analyses of variance (ANOVAs), self-construal and change consequences were factors in the design.

### Manipulation Checks

To assess whether our manipulation was successful, we first conducted a *χ*^2^ test on participants’ answer to the question about whether they wrote a description of how they function in individual or in group settings. A total of 83.15 % of the participants answered accurately, *χ*^2^ (1, *N* = 88) = 39.14, *p* < .001. A two-way ANOVA on our change consequence manipulation check revealed that participants in the individual change consequences condition (*M* = 4.13, SD = 1.44) felt more strongly that the change would affect their personal functioning than those in the group change orientation condition (*M* = 2.05, SD = 1.16), *F*(1, 85) = 55.46, *p* < .001, *η*_p_^2^ = .39). No other effects were found.

### Stress

To test our hypotheses, we first conducted a contrast analysis on our SBP measure. We specifically tested whether participants had higher SBP when there was a match between self-construal and change consequences than when there was no match. Contrast weights associated with this prediction were 1, −1, −1, and 1, respectively, with a ‘1’ assigned to the matching conditions, and a ‘−1’ assigned to the mismatching conditions (Rosnow and Rosenthal 1996). As control variables, we used SBP before the change, sex, and age as they are sometimes considered to influence later SBP (Hart et al. 2012). Confirming Hypothesis 1, the specified contrast was significant in the expected direction (*F*(1, 84) = 4.43, *p* < .05, *η*_p_^2^ = .05).

**Fig. 2:**
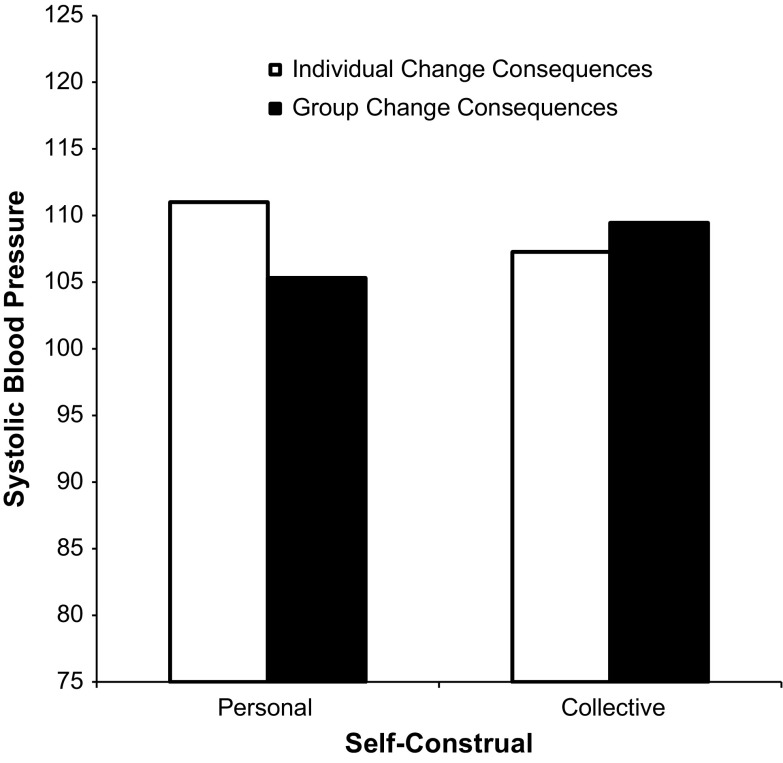
Systolic blood pressure as a function of self-construal and change consequences in Study 1

To gain more insight into the pattern of results, we then conducted a 2 × 2 ANCOVA on SBP (again controlling for SBP before the change, sex, and age). The ANCOVA revealed, as expected, a Self-Construal × Change Consequences interaction, *F*(1, 84) = 4.43, *p* = .035, *η*_p_^2^ = .04 (see Fig. 2). Simple main effects’ analyses revealed a marginally significant effect, indicating that participants in the personal self condition had higher BP when the change targeted the individual him or herself (M = 111.00, SD = 12.48) than when it targeted the group (*M* = 105.32, SD = 12.43), *F*(1, 84) = 3.37, *p* < .07, *η*_p_^2^ = .04. There was no indication that participants in the collective self condition responded with different SBP depending on the change consequences condition (*F*(1, 84) = 1.21, *p* = ns, *η*_p_^2^ = .01).

We also conducted an ANCOVA on DBP after the change. Again we used as control variables DBP before the change, sex, and age. As in Pollard (2001), no effects emerged.

## Method Study 2

### Participants and Design

A total of 135 employees from a diverse set of industries in the United States (81 male; *M*_age_ = 33.36, SD = 10.61) participated in our online scenario experiment.^2^ Respondents were randomly assigned to a 2 (Self-Construal: personal vs. collective) × 2 (Change Consequences: individual vs. group) between-subjects design. The 4 conditions comprised between 33 and 35 participants each. Only respondents holding a paid position with a minimum of 3 days a week were allowed to participate. Respondents’ job tenure was less than a year (16.3 %), 1–5 years (57.0 %), 6–10 years (12.6 %), or 11 or more years (14 %). Respondents with a higher education (i.e., bachelor’s degree or higher) made up 61.5 % of the sample. Most respondents had a white Caucasian background (74.8 %). Respondents were recruited using Amazon’s Mechanical Turk Website and were paid 45 US cents for their participation. Note that previous research has shown that data obtained with Mechanical Turk are at least as reliable as those obtained via traditional methods (Buhrmester et al. 2011; Paolacci et al. 2010).

### Procedure and Manipulations

Respondents read a scenario in which they were asked to imagine they were working, in a team setting, for a medium-sized organization that produced high quality products. Similar to Study 1, we then first introduced our *self*-*construal manipulation* (based on van Knippenberg et al. 2006, and Wisse and Rus 2012). In the *personal self* condition, respondents read that it was clear to them that they have a unique identity independent of their other team members. Consequently, they did not feel closely connected to the others. It was personal growth and development that was important to them. For instance, participants read “…*you feel good about yourself when you perform better than your colleagues, and when you talk about the team you usually say ‘they’ rather than ‘we.’ It is clear that at work you are motivated to achieve personal (rather than team) success. For you, it is particularly important to be able to strive for your personal goals*.” In the *collective self* condition, respondents read that it was clear to them that they had a lot in common with their other team members and that they are very much like them. Consequently, they did feel closely connected to the others. Moreover, collective growth and development were important to them. For instance, participants read “*…when someone praises your team, it feels like a personal compliment, and when you talk about the team you usually say ‘we’ rather than ‘they.’ It is clear that at work you are motivated to achieve team (rather than personal) success. For you, it is particularly important to be able to strive for team goals*.”

Next respondents learned that an organizational change was forthcoming. This change would affect what was prioritized and considered important in the organization. We told respondents in the *individual change consequences* condition that it was unclear whether in the new organization, they would still be able to function as they currently did, and whether there would be room for their personal skills and knowledge. The question was thus whether they, as individual employees, would be valued in the new organization. In contrast, we told respondents in the *group change consequences* condition that it was unclear whether in the new organization, it would still be possible for the team to function as it currently did, and whether there would be room for the team-based skills and knowledge. The question was thus whether the team as a whole would be valued in the new organization. Finally, respondents answered some questions, and then were thanked for their participation.

### Dependent Measures

All items had 5-point scales ranging from *strongly disagree* (1) to *strongly agree* (5). As a check of our self-construal manipulation, respondents answered a 2-item personal self-construal scale (e.g., “I see myself as an individual, separate from others,” “At work, I focus on my personal interests,” *α* = .91) and a 2-item collective self-construal scale (e.g., “I see myself as part of the team,” “At work, I focus on the team’s interest,” *α* = .97). To check our change consequences manipulation, we asked respondents to indicate their level of agreement with the statement that the change will mainly affect the appreciation for personal skills, knowledge, and behaviors, and with the statement that the change will mainly affect the appreciation for team-based skills, knowledge, and behaviors.

*Anticipateduncertainty* was measured with 6 items (*α* = .94.). Items were developed to assess general feelings of uncertainty. Examples are “In this situation I would feel very uncertain,” “I would wonder what the future would bring,” and “In this situation I would feel quite confident (*R*).”

*Anticipatedstress* was measured with the 30-item Perceived Stress Questionnaire (Levenstein et al. 1993). This scale has high construct validity and correlates with the development of physical illness. It emphasizes cognitive perceptions about stress (i.e., descriptions of the situation) rather than emotional states or life events, which fits the methodological approach in this study (i.e., a scenario experiment). Items were slightly adapted to fit the context. Examples are “In this situation I would be irritable or grouchy,” and “In this situation I would have many worries” (*α* = .88).

## Results Study 2

### Manipulation Checks

To assess whether our manipulation was successful, we first conducted 2 × 2 ANOVAs on our manipulation checks. The ANOVA on the personal self scale revealed that respondents in the personal self conditions (*M* = 4.43, SD = 0.64) scored higher on the scale than those in the collective self conditions (*M* = 2.14, SD = 0.96), *F*(1, 131) = 267.54, *p* < .001, *η*_p_^2^ = .67. Additionally, the ANOVA on the collective self scale showed that respondents in the collective self conditions (*M* = 4.71, SD = 0.48) scored higher on the scale than those in the personal self conditions (*M* = 2.03, SD = 0.85), *F*(1, 131) = 493.39, *p* < .001, *η*_p_^2^ = .79. A subsequent ANOVA revealed that respondents in the individual change consequences condition (*M* = 4.00, SD = 1.13) felt more strongly than those in the group change consequences condition that the change mainly affected their personal functioning (*M* = 2.20, SD = 1.01), *F*(1, 131) = 94.55, *p* < .01, *η*_p_^2^ = .42). Finally, ANOVA indicated that respondents in the group change consequences condition (*M* = 4.25, SD = 0.80) felt more strongly than those in the individual change consequences condition that the change mainly affected team functioning (*M* = 2.56, SD = 1.25), *F*(1, 131) = 86.76, *p* < .01, *η*_p_^2^ = .40). No other effects than the ones reported were found.

### Anticipated Stress

To test our first hypothesis, we again first conducted a contrast analysis on our stress measure. We specifically tested whether respondents anticipated more stress when there was a match between self-construal and change consequences than when there was no match (contrast weights were 1, −1, −1, 1, with a ‘1’ assigned to the matching conditions, and a ‘−1’ assigned to the mismatching conditions; Rosnow and Rosenthal 1996). Gender and age were included as control variables (cf., Dahl 2011). Confirming Hypothesis 1, the specified contrast was significant in the expected direction (*F*(1, 131) = 4.88, *p* < .05, *η*_p_^2^ = .04).

To further scrutinize the pattern of results, we then conducted a 2 × 2 ANCOVA on our stress measure and included gender and age as control variables. This revealed a main effect of change consequences, *F*(1, 131) = 9.14, *p* = .005, *η*_p_^2^ = .06, with respondents in the individual change consequences (*M* = 2.69, SD = 0.64) reporting more stress than those in the group change consequences (*M* = 2.36, SD = 0.60). We also found a Self-Construal × Change consequences interaction, *F*(1, 131) = 4.19, *p* < .05, *η*_p_^2^ = .03. Respondents in the personal self condition felt more stress when the change targeted the individual (M = 2.81, SD = 0.62) than when it targeted the group (*M* = 2.26, SD = 0.66), *F*(1, 131) = 13.13, *p* < .05, *η*_p_^2^ = .09. No effect of change consequences was found for respondents in the collective self condition (*M*_individual consequences_ = 2.55, SD = 0.65 vs. *M*_group consequences_ = 2.47, SD = 0.51, *F*(1, 131) = 0.45, *ns*). Similar to the results in Study 1, these findings indicate that people whose personal (vs. collective) self is salient are particularly likely to react strongly to change that targets their salient level of self-construal.

### The Role of Uncertainty

We predicted that uncertainty would mediate the relationship between the interaction of self-construal and change consequences on feelings of stress. To test this second hypothesis properly, we ran a moderated mediation analysis using bias-corrected bootstrapping (Hayes 2013). This procedure has three steps in which we controlled for gender and age (see Table 1 for relevant statistical details). In Step 1, a regression analysis was conducted to test whether self-construal, change consequences, and their interaction influence uncertainty (mediator model). As expected, the self-construal × change consequences interaction significantly influenced uncertainty. In Step 2, a regression analysis was conducted wherein anticipated stress was regressed on change consequences, self-construal, their interaction, and uncertainty (dependent variable model). This revealed that uncertainty indeed influenced anticipated stress. It also revealed that adding uncertainty to the design left the self-construal × change consequences interaction insignificant. In Step 3, we tested the conditional indirect effects of change consequences via the mediator on the dependent variable at different levels of self-construal. This confirmed that change that affected individual functioning resulted in higher levels of anticipated stress than change that affected group functioning via uncertainty for people whose personal self was salient. The type of change did not influence anticipated stress via uncertainty for employees whose collective self was salient.

**Table 1:** Regression results for the conditional indirect effects of Study 2

Predictor	Mediator variable model (DV = uncertainty)		
	*b*ª	SE	*t*(133)
Constant	5.22	0.74	7.04**
Age	0.00	0.01	0.16
Gender	−0.00	0.14	−0.00
Change consequences	−0.64	0.45	−1.43
Self-construal	−1.40	0.45	−3.13**
Change consequences × self-construal	0.74	0.28	2.63**

Predictor	Dependent variable model (DV = stress)		
	*b*ª	SE	*t*(132)
Constant	0.31	0.44	0.69
Age	−0.00	0.00	−0.97
Gender	−0.11	0.07	−1.51
Uncertainty	0.56	0.04	12.55**
Change consequences	0.03	0.23	0.11
Self-construal	0.15	0.24	0.65
Change consequences × self-construal	0.02	0.15	0.14

	Conditional indirect effects at values of the moderator			
	Effect	Boot SE	BootLLCI	BootULCI
Personal self	0.47	0.12	0.25	0.73
Collective self	0.06	0.10	−0.15	0.26

^a^Unstandardized regression coefficients

* *p* < .05; ** *p* < .01

Thus, employees whose personal self is salient anticipate more stress from change that affects individual-level functioning than from change that affects group-level functioning, and this effect is mediated by anticipated uncertainty.

## Conclusion Study 1 and 2

Studies 1 and 2 provide first empirical evidence that individuals react differently to different types of change (a change that has direct consequences for individual functioning vs. a change that has direct consequences for group functioning), depending on the level of inclusiveness at which their self is construed. Although we found that a closer match between the two appears to result in more stress, it is important to note that we only found effects of the specific type of change for people whose personal self was salient and not for people whose collective self was salient. Apparently people whose personal self was salient reacted more strongly to change that threatened the enactment of their self-identity than people whose collective self was salient. One theoretical explanation for this is that the personal self is the more fundamental level of self-representation (see Sedikides and Gaertner 2001), particularly in Western societies (Oyserman and Lee 2008). In line with this notion, there is some evidence to show that people are more displeased after threats to the personal self than to the collective self, and that a threat to the personal self induces more protective strategies than a threat to the collective self (Gaertner et al. 1999). In addition, it has been found that factors that promote behavior congruent with one’s internal state, have a stronger influence on individuals whose personal self is salient than on those whose collective self is salient (Wisse and Rus 2012). Finally, when people feel their collective self at work to be threatened by a particular change, they may find refuge in their personal self and distance themselves from their group (e.g., although the future of my work group is unsure, I am confident that there is still room for my personal qualities and competencies). However, when the personal self is threatened and the change targets one’s individual identity, it may be difficult to find a suitable alternative identity within the same organizational context (cf., Petriglieri 2011). The results of Study 2 also indicate that uncertainty acts as a mediator in the combined effects of change consequences and self-construal on anticipated stress.

To assess whether we could confirm our main findings in the field, we conducted a follow-up study among employees who were working in an organization that was in the midst of a change process (Study 3). In this third study, we further investigate how change may be related to uncertainty and stress for employees with different strengths of personal self. Based on our findings so far, as well on our previous theorizing, we expect that change that threatens individual functioning will be experienced as more stressful to the extent the employees personal self is salient, and that these effects can be explained by feelings of uncertainty.

## Method Study 3

### Participants and Procedure

We approached 339 employees of an internationally oriented Dutch organization, active in the chemical industry and worldwide market leader in its branch, to fill in the online questionnaire. At the time of the survey, the organization was in the midst of a restructuring process to reduce costs. A total of 276 employees filled the survey out completely (82.0 % response rate). Of the employees, 14 % had worked up to 2 years for the organization, 15.8 % between 2 and 5 years, 20.1 % between 5 and 10 years, 23.7 % between 10 and 20 years, and 26.5 % longer than 20 years. Moreover, 11.5 % of the employees had a supervisory position. Company policies prohibited to include items about age and gender in the survey; HRM provided information about employees age (average = 41 years; 16 % under 35, 42 % over 45), and gender (78 % male). Because people were approached at work, we were urged to keep the survey short and to the point.

### Measures

All responses were assessed using a 5-point Likert-type scale (1 = *strongly disagree*, 5 = *strongly agree*). Overall, the reliability of the scales used was good (*α* > .80). All items of the predictor measures can be found in the “Appendix” section. The *salience of employees’ personal self* was measured with 8 items that we based on Selenta and Lord (2005) and Wisse and Rus (2012). To measure the extent to which employees perceived the change to be consequential for the individual or for the group, we developed a 4-item scale and a 5-item scale. We used two items to measure *uncertainty* caused by the change. To measure *stress*, we used the Maslach Burnout Inventory-General Survey (Schaufeli et al. 1996), which was originally developed to measure occupational stress-related burnout. We only used a 5-item emotional exhaustion sub-scale, because this sub-scale represents the basic individual stress dimension of burnout (Maslach et al. 2001) and is the most stable dimension (Brenninkmeijer and van Yperen 2003). Emotional exhaustion is predictive of the scores on the other two other sub-scales (e.g., cynicism and professional efficacy), which are more closely tied to behavioral burnout responses (Bakker et al. 2002). Sample items are “I feel burned out from my work,” and “I feel tired when I get up in the morning and have to face another day on the job.”

#### Covariates

The 5 categories of tenure were coded 1 (up to 2 years) to 5 (longer than 20 years). Employees who did not hold a supervisory position were coded (0) and employees who did hold a supervisory position were coded (1). To rule out the possibility that effects of the salience of personal self were induced by (perceived) relational attachment or investment in or of the organization or fellow employees, we included two control variables, namely perceived organizational support and turnover intentions. Theoretically based on the social exchange approach to organizational behavior (Gouldner 1960), perceived organizational support (Eisenberger et al. 1986) reflects a general perception of the extent to which the organization values people’s contributions and cares about their well-being. As acknowledged in a vast amount of empirical works (see van Knippenberg et al. 2015 for state-of-the-art literature), perceived organizational support has shown to be highly indicative for the relational significance people attach to the organization. In addition, turnover intentions are seen as indicative for a general sense of disrespect from fellow group members and for the lack of quality of the psychological link between them (Sleebos et al. 2006a, b).

## Results Study 3

Confirmatory Factor Analysis (EQS 6.1 for Windows; Bentler and Wu 2004) supported the distinctiveness of our five study variables. Fit-indexes showed a satisfactorily fit *χ*_309_^2^ = 589.203, *p* = .001, RMSEA = .057, NNFI = .91, CFI = .92. This five-factor fit was superior to the fit of the next most likely model (Δ*χ*^2^ = 83.47, *p* < .001), a four factor model where the highest correlated factors ‘individual change consequences’ and ‘group change consequences’ (*r* = .66) were included as one factor. Table 2 shows means, standard deviations, zero-order Pearson correlations, and Cronbach’s alphas for the study variables.

**Table 2:** Descriptive statistics, Cronbach’s alphas, and intercorrelations for the Study 3 variables

Variables (*N* = 276)	Mean	SD	1	2	3	4	5	6	7	8	9
1. Personal self	2.90	0.63	(.81)								
2. Individual change consequences	3.15	0.91	.11	(.85)							
3. Group change consequences	3.64	0.80	.11	.66**	(.80)						
4. Uncertainty	2.96	1.06	.07	.52**	.39**	(.80)					
5. Stress	3.04	1.53	.11	.46**	.41**	.35**	(.96)				
Covariates											
6. Position	–	–	.06	.06	−.02	−.17**	.02	–			
7. Tenure	–	–	.00	.23**	.23**	.10	.25**	.02	–		
8. Perceived organizational support	2.67	0.82	.02	−.40**	−.41**	−.27**	−.31**	.08	−.19**	(.93)	
9. Turnover intentions	2.40	0.97	.06	.26**	.26**	.10	.37**	.03	.07	−.41**	(.91)

Cronbach’s alphas are depicted on the diagonal

* *p* < .05; ** *p* < .01 (two-tailed significance)

### Perceived Stress

**Fig. 3:**
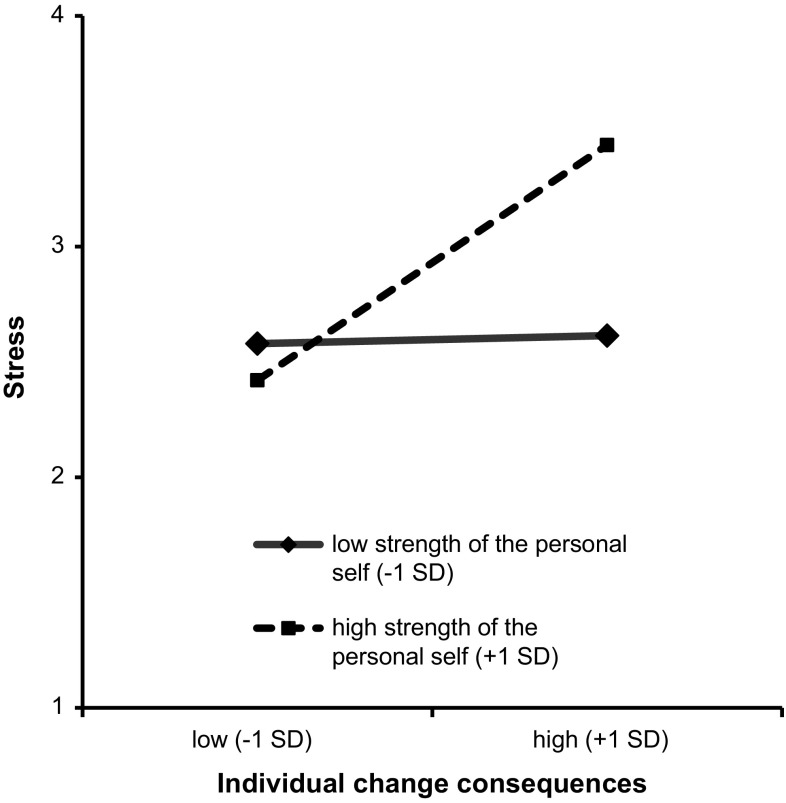
Perceived stress as a function of personal self-construal and individual change consequences in Study 3

We predicted that the salience of the personal self and the experience of individual-level change would interact in such way that particularly when employees’ personal self is salient, individual change consequences would be related to stress. To test this hypothesized moderation accurately, we relied on a procedure suggested by Hayes (2013). We controlled for tenure, position, perceived organizational support, and turnover intentions (see Table 3 for relevant statistical details). We found no main effects. However, in line with our hypothesis, we did find that the interaction term of personal self-construal and perceptions of individual consequences of change significantly influenced stress. We tested the conditional direct effects of individual change consequences on the dependent variable (stress) at different levels of personal self-construal (see Fig. 3). Bootstrapping (5000 samples) confirmed that the direct effect of individual change consequences on stress was significant for high salience of the personal self (*b* = .70, *p* < .001), but not for low salience of the personal self (*b* = .24, *p* = ns.). Note that the interaction term of personal self-construal and perceptions of collective consequences of change did not have a significant effect on stress.

**Table 3:** Regression results for the conditional effects of Study 3

Predictor	Dependent variable model (DV = stress)		
	*b*ª	SE	*t*(266)
Constant	.83	1.67	.50
Position	−.08	.28	−.29
Tenure	.16**	.06	2.69
Perceived organizational support	−.04	.11	−.34
Turnover intentions	.39	.09	4.46
Personal self	−.45	.53	−.85
Individual consequences of change	−.59	.47	−1.25
Group consequences of change	.66	.54	1.23
Personal self × individual change consequences	.36*	.16	2.22
Personal self × group change consequences	−.16	.19	−.86

	Conditional effect of *X* on *Y* at values of the moderator		
	Effect	SE	*t*(266)
Personal self (high = +1SD)	.70**	.17	4.10
Personal self (low = −1SD)	.24	.14	1.70

^a^Unstandardized regression coefficients

* *p* < .05; ** *p* < .01

### The Role of Uncertainty

Similar to Study 2, we predicted that for employees with a salient personal self-construal, the experience of individual-level change would be related to uncertainty, which, in turn, would be related to stress. To properly test this hypothesis, we ran a moderated mediation analysis using bias-corrected bootstrapping (Hayes 2013; see Table 4), and we controlled for tenure, position, perceived organizational support and turnover intentions. In Step 1, we found that the interaction term of personal self-construal and perception of individual consequences was significantly related to uncertainty (mediator model). In Step 2, we tested whether uncertainty was related to stress (dependent variable model), and the results show that this was indeed the case. In Step 3, we tested the conditional indirect effects of change consequences via the mediator on the dependent variable (stress) at different levels of personal self-construal. Bootstrapping (5000 samples) confirmed that the indirect effect of individual change consequences on stress through uncertainty is consistently significant for high and low levels of the personal self. Given that the effect of individual change consequences on stress was only significant for high salience of the personal self, we may conclude that the perception that the change has individual consequences is related to stress for employees whose personal self is salient via feelings of uncertainty.

**Table 4:** Regression results for the conditional indirect effects of Study 3

Predictor	Mediator variable model (DV = uncertainty)			Dependent variable model (DV = stress)		
	*b*ª	SE	*t*(266)	*b*ª	SE	*t*(265)
Constant	3.15**	1.16	2.71	.17	1.68	.10
Position	−.74**	.19	−3.84	.07	.28	.26
Tenure	−.02	.04	−.50	.16**	.06	2.78
Perceived organizational support	−.09	.08	−1.13	−.02	.11	−.18
Turnover intentions	−.07	.06	−1.12	.40	.09	4.65
Uncertainty	–	–	–	.21*	.09	2.38
Personal self	−.56	.37	−1.51	−.33	.53	−.63
Individual consequences of change	−.09	.33	−.26	−.57	.47	−1.22
Group consequences of change	.15	.37	.39	.63	.53	1.18
Personal self × individual change consequences	.23*	.11	2.00	.32	.16	1.93
Personal self × group change consequences	−.04	.13	−.28	−.16	.19	−.83

	Conditional indirect effects at values of the moderator			
	Effect	Boot SE	BootLLCI	BootULCI
Personal self (high = +1SD)	.15	.07	.03	.30
Personal self (low = −1SD)	.09	.05	.02	.22

^a^Unstandardized regression coefficients

* *p* < .05; ** *p* < .01

## General Discussion

This study investigated self-construal and change consequences as antecedents of stress reactions to change. We set up a structured approach to test the models’ viability by conducting one experimental laboratory study, one scenario study and one field study. Confirming our hypotheses, self-construal interacted with change consequences to predict physiological stress responses (Study 1), anticipated psychological stress (Study 2), and perceived psychological stress (Study 3).

The results of this research provide an explanation for why there are considerable inter-individual differences in the amount of stress that employees experience during change. The present research suggests that the dynamics between individual characteristics and the context of the change need to be taken into account. Specifically, our study shows, in conjunction with previous theoretical and empirical studies, that the extent to which the change infringes on salient aspects of the self-concept is of major importance for people’s responses to change. Importantly, however, our findings suggest that the responses of people whose personal self is salient are more affected by whether the change affects individual-level functioning or group-level functioning than is the case for people whose collective self is salient. Our findings underscore the fundamental importance of the self-concept for individuals and show that a discontinuity in the enactment of an identity may be highly stressful, perhaps because it makes people uncertain.

### Future Research

We would like to draw attention to three more issues that could fruitfully be addressed in future research. First, future research could further explore the role of self-construal in people’s reactions to change. Specifically, although previous research alluded to the possibility that the integrative effect of collective self and group change consequences might be weaker than the integrative effect of personal self and individual change consequences, we did not necessarily anticipate the former to have as little effect as it did in the present study. Apart from the reasons, we already offered in the discussion section after Study 2, an additional reason for this lack of effect could be that both uncertainty and stress are individual difference variables that may be less suitable as outcomes measures for collective or group processes. Future research could investigate whether the combined effect of collective self and group change consequences may be more apparent on outcome measures such as group efficacy or collective self-esteem.

Second, whereas we focused only on the personal self and the collective self, future research may also focus on the relational self. The relational self implies a psychological merging of self and other, and is based on the individual’s roles in relationships with significant others, such as family, friends, colleagues, or supervisors (Brewer and Gardner 1996; Markus and Kitayama 1991; Sedikides and Brewer 2001). For the relational self, change that affects the dyadic interaction with significant others may have a particularly large impact.

Third, a process that also requires empirical attention is what has been called the ‘trickle-down effect.’ Our study is one of the few to differentiate between change that explicitly targets individual functioning and change that explicitly targets group functioning. Yet, in our study, we have not explained or tested empirically how group-focused changes and individual-focused changes may influence one another (cf., Datta et al. 2010; Whelan-Berry et al. 2003). For instance, change that is initiated at one level in the organization may have consequences for operations at another. Future research could explore this issue further.

Finally, the concepts of personal self-construal and collective self-construal are closely associated with the cultural psychological concepts of individualism and collectivism. These constructs summarize differences in how the relationship between individuals and societies is construed and whether individuals or groups are seen as the basic unit of analyses (Oyserman and Lee 2008). In individualistic cultures (such as in most Western countries), personal goals are placed ahead of collective goals and societies are seen to exist to cater to the needs of individuals. In collectivistic cultures (such as in many non-Western and Asian countries), collective goals are placed ahead of personal ones and individuals are expected to make an effort to fit into society (Markus and Kitayama 1991; Triandis 1995). As a consequence, in individualistic cultures, schema for the personal self are more readily accessible, while in collectivistic cultures, schema for a collective self are more readily accessible (Markus and Kitayama 1991). Pekerti and Kwantes (2011) showed that cultural background indeed affects peoples’ self-construal. They also showed that self-construal, in turn, predicted peoples’ perceptions of organizational events. Notably, these findings could point to a limited generalizability of our findings. The samples in our study came from (mostly) individualistic cultures, making the participants more naturally inclined toward the salience of the personal self. As a consequence, it may for instance be that the collective self-construal inductions were less effective than the personal self-construal inductions. Future research could investigate whether in individualistic countries, where a personal self-construal is more likely, organizational change that relates to the functioning of individuals leads to more stress than change that relates to the functioning of the group, while in collectivistic countries, where a collective self-construal is more likely, organizational change that relates to the functioning of groups leads to more stress than change that relates to the functioning of individuals.

### Strengths and Limitations

As with every study, the present study has its strengths and limitations. One strength is that by conducting a laboratory experiment, a vignette study and a field study, we adopted a multiple-study, multiple-method approach in which the strengths of one method may compensate for any weaknesses in others (Eid and Diener 2006). The advantage of using experiments is that it makes causal inferences possible and may increase confidence in the internal validity of the study. In addition, the experimental design facilitated the measurement of physiological responses (BP) in Study 1. Note that the SBP of our respondents was on average on the lower side. This may have been due to the fact that our respondents were young, highly educated, and predominantly female (Loucks et al. 2011; Reckelhoff 2001). Higher average BPs might be found in a more heterogeneous sample of employees.

Of course, we are aware of the potential pitfalls of using experiments to investigate change. For instance, our laboratory experiment (Study 1) could be criticized for its artificial character, and our scenario study (Study 2) could be criticized for assessing people’s responses to a hypothetical situation. Although we took special care to achieve a high degree of experimental realism (Study 1) and mundane realism (Study 2), the findings generated in the experimental environment provide no evidence that the same relationships actually exist outside the laboratory (Goodwin et al. 2000). Study 3 may alleviate that concern as it shows that these relationships may indeed be observed in the field. For this study, however, the cross-sectional single-source design may be deemed suboptimal; one reason being that no causal inferences can be made with such a design. Note that common method variance cannot account for interactions in regression (McClelland and Judd 1993; Siemens et al. 2010), and as such it does not pose a threat to the validity of our results.

### Practical Implications

Although implications for practice should still be seen as tentative, our study suggests that the organization may take steps to ensure a sense of continuity of identity. Some suggestions about how to realize that employees experience such a sense of continuity of identity have been made. For instance, it has been argued that if observable continuity (i.e., ‘objective’ indicators of continuity such as maintaining distinctive sub-units in a merger) is lacking, the organization may try to instill in employees *projected continuity*, or the expectation that the future identity will be linked to the past and present identity (Ullrich et al. 2005). Leadership may play an important role in this process. Shamir et al. (1993), for instance, suggest that charismatic leaders are effective because they are able to instill a sense of self-consistency and continuity in employees (cf., Bono and Judge 2003). Evidently, more research on the role of change agents as agents of continuity is warranted (van Knippenberg and Hogg 2003). However, we hope that our study will be a useful step in reducing the chance that people come to regard employee stress as an inescapable part of change and in helping them to focus instead on opportunities to manage change effectively.

### Conclusion

Employees differ in the amount of stress they experience as a result of change. We have argued that it is important to recognize that reactions to change may be informed by employees’ self-concept, and to acknowledge that not all changes are the same,—they may differ in terms of the consequences they have for groups or for individuals. Our findings suggest that knowledge of how people react to change can be furthered by taking into account the interplay between individuals’ self-representation and contextual variables. We hope that the findings presented in this study may inspire future research and inform practitioners seeking answers as to how to ensure effective implementation of organizational changes.

## Appendix

### Measurement Predictor Variables Study 3

**Uncertainty**
1. The reorganization makes me feel uncertain.
2. As a result of the reorganization, I have more doubts.

**Personal self-construal**
1. I have a strong need to know how I stand in comparison to my colleagues.
2. I differentiate myself from my colleagues.
3. At work, I focus on the extent to which the decisions made are beneficial to me personally.
4. I often compete with colleagues at work.
5. At work, I especially exert myself if this can bring me personal success.
6. I feel best about myself when I perform better than my colleagues.
7. I just work to realize my own personal goals.
8. I thrive on opportunities to demonstrate that my abilities or talents are better than those of my colleagues.

**Individual change consequences**
1. Because of the reorganization, I will no longer be the person I always was.
2. Because of the reorganization, my personal goals at work will change.
3. The reorganization has consequences for my personal functioning.
4. Because of the reorganization, the goals and targets of my job will change.

**Collective change consequences**
1. Because of the reorganization, my team will no longer be what it always was.
2. Because of the reorganization, the atmosphere in my team will change.
3. Because of the reorganization, norms and values within my team will change.
4. The reorganization has consequences for my team’s functioning.
5. Because of the reorganization, the goals and targets of my team will change.

## Footnotes

1 Note that some data were lost for instance because the monitor was not properly placed on participants’ arm, or because participants failed to follow recording instructions. These participants were removed from the dataset.

2 Eight respondents were not included in the analyses because they were well above retirement age (>70 years ), or because they spent less than 4 s. on reading the text we used to manipulate our variables of interest.
